# “The Art of Loving”: A Psychobiographical Perspective on Erich Fromm’s Life and Love Concepts

**DOI:** 10.17505/jpor.2026.29050

**Published:** 2026-03-26

**Authors:** Lolo Jacques PN Mayer, Claude-Hélène Mayer

**Affiliations:** 1 Department of Psychology, University of London, London, UK. E-mail: lolomayer@gmx.net Orcid ID: https://orcid.org/0009-0005-6854-2724; 2 Department of Industrial Psychology and People Management, University of Johannesburg, Johannesburg, South Africa, E-mail: cmayer@uj.ac.za Orcid ID: https://orcid.org/0000-0002-9445-7591

**Keywords:** love as art, human connection, human relationships, emotional dimensions of love, love and culture, types of love, Erich Fromm

## Abstract

Erich Fromm (1900–1980) was a sociologist, psychoanalyst and a social and human philosopher of German-Jewish origin who fled from Germany via Geneva to the United States of America in 1934. He became globally known as the author of the book *The Art of Loving* (Fromm, 1956) and stated that the only way to lead a sane and satisfying human existence is through love. This psychobiographical study aims to capture the theme of love throughout the life of Erich Fromm and within selected creative works. The research methodology used is qualitative in nature and applies a psychobiographical study approach. Psychobiographies explore the lives of extraordinary individuals through psychological theories. This study focuses on the life of Fromm as a single purposefully selected individual. Data was collected from literature search in the public domains and analysed through content analysis. The study used a five-step-content analysis process of familiarisation with the data, coding and categorisation, analysis, data reconstruction and presentation. Findings trace the development of the concept of love in the life and work of Fromm. Fromm viewed love as an “art” which needs to be learned, and requires discipline, focus, concentration and patience. It is an active skill that needs effort and understanding. Different forms of love exist, such as the love of God, the love within family (motherly love, brotherly love), love in love relationships (erotic love) and the love for the self (self-love). The four core aspects of love are care, responsibility, respect and knowledge. In this article, we draw selected conclusions on how Fromm’s concept of love may have grown from his lived experience. These findings and conclusions might deserve more detailed analyses in future research.

*Love isn't something natural*.

*Rather it requires discipline,*

*concentration, patience, faith,*

*and the overcoming of narcissism*.

*It isn't a feeling; it is a practice*.

Erich Fromm (1956)

## 1. Introduction

Interdisciplinary love research has increased markedly over the past 60 years, in particular in the humanities, anthropology, sociology and history (Rokach, 2024). Berman (2024, p. 5) has recently provided new insights into the “paradox of love” by exploring historical and philosophical perspectives on love, exclusion, and potential for liberation. He points out that love, even in recent research, is a phenomenon that resists definition on the one hand although it is “commonly recognized and experienced” on the other hand (Berman, 2024, p. 5). The current article focuses on the conceptualisation of love during Fromm’s life and in selected writings to gain a deeper understanding of love as a self-transcendent force. It thereby interlinks with the new interest in Fromm’s work in the context of the revival of authoritarian political systems (Cortina, 2015, 2024; Durkin & Braune, 2020; Funk, 2022).

This psychobiographical study aims to capture the theme of love throughout the life of Erich Fromm and within selected creative works. To support this aim, three of his selected works (Fromm 1941, 1955, 1956), were analysed to examine his original contributions to love research in the 20th century. Thereby, we aim to explore further how his life experiences and his theoretical explorations of love were intertwined. To fulfil the aim, we respond to the research question: What is love in the context of Erich Fromm’s life and selected works? By responding to this research question, this article contributes to psychobiographical literature on the development of Fromm’s love concept in the context of his personal, lived experiences.

European love research in the 20th century was strongly influenced by discourses concerning love and its complex connection with identity development, an exploration of the sense of belonging and by the development of romantic and courtly love (Brooks, 2019; Fromm, 1956; Mayer & Vanderheiden, 2021, 2023; Passerini, 2010). Notably in post-war contexts, researchers in Western industrialised countries started to explore love in terms of desire and sexuality and questioned how love and culture were interlinked (Reddy, 2010; Smieja et al., 2025). European scholars declared at that time that love was uniquely European (Ellena, 2010) and applied a Eurocentric, imperialised and colonial view of love from the 19th century onwards. Later research, however, has shown that while love is a universal concept, it is culturally determined and needs to be explored from culture-specific perspectives (Karandashev, 2017; Mayer & Vanderheiden, 2025a, 2025b).

Fromm, who lived in different cultural contexts – Germany, Switzerland, the United States of America (USA) and Mexico – included different views on love in his conceptualisations. He did not claim that love was a European concept but rather emphasised its universality and described love as an emotion that existed not only between individuals, but also between individuals and the society. He harshly criticised modern societies for creating loneliness, disconnectedness and isolation and called for a radical humanism that focuses on flourishing in a humane world (Cortina, 2024; Fromm, 1956). In this respect, Fromm can be viewed as a pioneer of contemporary societal criticism regarding the problem of diminishing authentic relationships and increasing narcissism and ego-orientation in a digitalised world (Funk, 2024). Finally, Fromm offered a vision of love as an emotion that can be influenced, developed and stimulated. In so doing, he moved away from a somewhat romanticised image of love (Fromm, 1958).

## 2. Conceptualisations of Love as a Self-Transcendent Emotion

In this article, we focus on the life and work of Erich Seligman Fromm, a German-Jewish sociologist, social psychologist and psychoanalyst who lived from 1900 to 1980 (CHMC, 2022). He was also a human philosopher, becoming globally known as the author of the book *The Art of Loving* (Fromm, 1956). He was one of the most influential thinkers on love in the 20th century and strongly influenced love research (Friedman, 2014; Fromm, 2020, 2024; Spiers, 2024). A major realisation, as highlighted by Berman (2024) was that love is commonly experienced, but not theorised or developed. Fromm developed aspects of his theories based on his personal experiences of love between individuals and in society and was one of the first European psychologists and psychoanalysts to recognise the importance of love as a transcendent force (Fromm, 1958).

**Figure 1:**
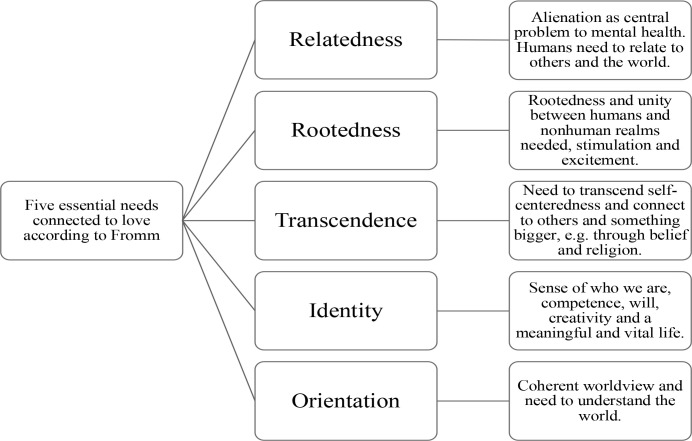
Fromm’s essential needs connected to love (authors’ own construction)

Fromm (1958) argues that identity, love and transcendence are important for individuals. These individual concepts are usually influenced by their families, the cultural context, the society, and by being human (Elsayed, 2024; Fromm, 1958; Jankowiak & Nelson, 2026). His understanding of love permeates both his personal philosophy and his broader critiques of modern society (O’Donnokoé et al., 2025) and connects to five fundamental human needs (see Figure 1), these being relatedness, rootedness, transcendence, identity and orientation (CHMC, 2022; Fromm, 1959; Hunt, 2018; Kühn, 2024; Wilde, 2016).

One major aspect of the conceptualisation of love in Fromm’s perspective is that it is viewed as a self-transcendent emotion, highlighting that an individual transcends their individual needs and desires towards the other (Fromm, 1956; Stellar et al., 2017). Self-transcendence is defined as a development that goes beyond personal growth and individual goals; it is something that contributes to a greater good, to others and to society (Fromm, 1956; Jan, 2024).

This self-transcendence could be experienced and expressed through feelings of unity and oneness and can be human-orientated but may also be associated with individual experiences of spirituality or nature (Fromm, 1956; Yaden et al., 2017; Yaden & Newberg, 2022). Love becomes transcendent, especially when experienced as unconditional, and plays an important role in the context of personal growth, development and personality (Jan, 2024). Self-transcendent love connects to a greater purpose; it is holistic and generous, goes beyond the ego, connects to the other and is grounded in authentic self-love (Conn, 1998).

## 3. Psychobiography as a Research Methodology

This study uses a psychobiographical case study design (Elms, 2007). It is qualitative in nature and explores love in the life of a single individual, Erich Fromm, and in three of his selected writings. The case study aims to capture the theme of love throughout different phases of Fromm’s life and the creative works that are especially concerned with elaborating on the topic of love as a self-transcendent emotion. This psychobiography is person-centered, focusing on Fromm’s unique love concept while providing a holistic description and understanding of love across the lifespan of this specific individual. The authors thereby follow Carlson’s (1971) call to use an idiographic approach which focuses on the unique, whole individual rather to overemphasis general laws. Further, this article builds onto other research that integrates biographical data and person-focused methods to develop richer personality theories (Carson, 1988). According to McAdams (2005), psychobiography is the art of understanding a notable individual’s life, usually through psychological theory. In this psychobiography, however, we do not apply an external psychological theory but instead aim to describe the theory of love which Fromm developed throughout his life and writings. Psychobiography as a research method and an investigative tool has been discussed previously and this study relies on previously discussed theoretical papers (Köváry, 2011).

The psychobiography uses a hermeneutical research paradigm (Dilthey, 2003) and applies parts of Runyan’s (2005a,b) concept of historical interpretative psychology: on the one hand this paper uses Runyan`s approach to focus on an individual life within its historical and social contexts; on the other hand Runyan (2005a,b) points out the importance of methodological rigor, consistency and comprehensiveness. It integrates well with Dilthey’s (2003) hermeneutical approach which also supports the analysis of the individual person within its sociocultural context.

### 3.1. Sample and sampling technique

Erich Fromm was purposefully chosen as a subject of research since he is one of the most influential writers on love in 20th century Europe.

In contemporary qualitative research and particularly in psychobiography, researchers declare their positionality with regard to the subject’s life (Mayer et al., 2021; McAdams, 2023), to address bias and position towards the subject transparently and openly. In this case, the authors are a young Black male South African psychology student who is studying in England, and a middle-aged white female South African-based German professor of psychology, both of whom have an interest in European philosophy and love research. For the research project, the authors have applied psychobiographical research methodology to a white male psychologist and philosopher who lived in 20th century Europe, the USA and Mexico (Silva-Gracía, 1992). The authors aim to deeply understand Fromm by empathetically putting themselves in the perspective of a man who lived in different sociocultural surroundings. Both of the authors personally identify with living away from their home country, with being influenced by various and diverse sociocultural influences and experiences, and with Fromm’s interest in developing psychological and philosophical concepts.

### 3.2. Data collection and analysis

The data collection is anchored in Allport’s (1961) approach to the use of first- and third-person documents for analysis. For example, as first-person documents, the authors used Fromm’s articles and books (1939, 1941, 1947, 1955, 1956), autobiographical-based literature (Fromm, 2024), interview scripts and statements (from newspapers, journals, internet sources), film documentaries and videos. For the analysis of Fromm’s literary and creative works, three books were chosen to explore his love concepts in depth, namely *Escape from Freedom* (1941), *The Sane Society* (1955) and *The Art of Loving* (1956). These works were purposefully selected since they deal predominantly with love as a topic. With regard to third-person documents, the authors used selected previously written biographical accounts (Akrap, 2011; Burston, 1991; Friedman, 2014; Funk, 2000, 2006, 2022; Hardeck, 2005; Hausdorff, 1972; Hornstein, 2005; Lévy, 2002; McLaughlin, 2023; Thompson, 2009; Wehr, 1990; Werder, 1987).

The authors used content analysis to explore the texts (Mayer, 2011; Terre Blanche et al., 2006), to identify key issues in the dataset, especially focusing on love in the life of Erich Fromm through a five-step process including familarisation of data, leading into a process of hermeneutic interpretation and reconstruction of data. The data reconstruction is viewed as an interactive researcher–researched process (Mayring, 2014). The authors went through several rounds of intra- and inter-subjective validation processes (Yin, 2009) to explore the dataset described above, to verify the findings. The only predefined category during this content analysis process (Streib, 2005) was the category of love.

### 3.3. Qualitative quality criteria, ethics and limitations

Qualitative research criteria were defined to maintain the standards of this research study in terms of meaningfulness and to make the study confirmable, credible, accurate and to include trustworthiness, credibility, dependability, transferability and confirmability (Gummesson, 2000; Lincoln & Guba, 1985; Miles & Huberman, 1994; Sinkovics et al., 2008).

This psychobiography follows ethical principles, as discussed in recent psychobiographical discourses (Mayer & Kőváry, 2019), applying them throughout the research process (Ponterotto & Reynolds, 2019). The researchers only used publicly available sources. Consequently, the ethical risk was minimised and “postmortem privacy rights” and next-of-kin-rights were maintained (Ponterotto & Reynolds, 2019). Furthermore, because the authors chose a deceased subject, ethical challenges regarding the choice of the living subject were overcome (APA, 1977).

With regard to limitations, the study is demarcated by its theoretical and methodological framework (Mayer, 2025), the sociocultural and disciplinary background of the researchers, their personal, gender and sociocultural biases and their abilities to reflect, change perspectives, organise, analyse and reorganise data.

## 4. Love in Erich Fromm’s Life, Relationships and Writing

Erich Fromm’s personal life shaped his understanding of love in different ways, such as his relationship to his parents, being married three times, as well as the socio-political situation in Germany during his childhood, adolescence and young adulthood (Boeree, 2006). According to Friedman (2016), Fromm was a highly complex individual with varied visions and “lives” which influenced his radical humanism. Further, his humanism and his love concepts were strongly influenced by his “personal motivations, his character traits, positive and negative relational experiences influenced him at least as much as did more abstract philosophical, ethical, spiritual or political considerations” (Erös, 2016, p. 266). These individual, interpersonal and social considerations influenced Fromm’s definitions of love (Funk, 2019) and led to the idea that love is at best a self-transcendent emotion. Runyan (2006) has pointed out that the life of eminent psychologists often influences what they write in their major works and how they write about it. Further, Fromm`s interpersonal relationships influenced his ideas on love and contributed to develop the personal meanings of love throughout his life. This study supports Runyan’s (2013) argument that psychobiographies that include socio-cultural, historical and political aspects and analysis can help to create a deeper understanding of the individual:

### 4.1. Love in Fromm’s early life in Germany (1900–1922)

Fromm was born on 23 March 1900 in Frankfurt, Germany, as a single child to a Jewish middle-class family in which both of his parents were orthodox Jews (Burston, 1991). His father, Naphtali, was a businessman, and his mother, Rosa, was a housewife (Spartacus Educational, 2021). Fromm described his father as moody and emotionally distant (Burston, 1991) and also as “obsessive”, “very neurotic”, and “pathologically anxious” (Funk, 2000, p. 7). He felt that his father did not contribute well to his education, did not give him enough guidance and overwhelmed him with his anxiety (Funk, 2000).

Additionally, he described his mother as suffering from depression and as being overly religious (Burston, 1991). Funk (2006, p. 3) sketches the mother as funny and social (“lustige und gesellige Frau”), but also as a mother who held onto the love towards her son which made it difficult for Erich to develop and individuate. Friedman (2014) points out that Fromm carried the depressive mood of his mother throughout his life.

**Figure 2:**
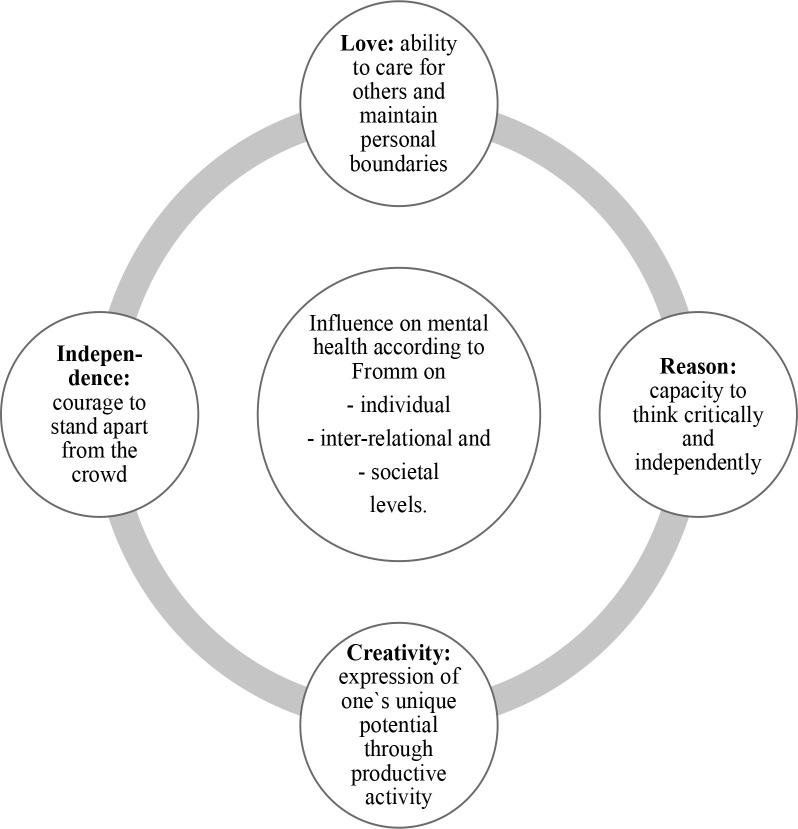
Mental health and love in Fromm’s conceptualisations (authors’ own construction).

In later years, Fromm himself described the marriage of his parents as unhappy, the father as extremely distant and the mother as overprotective (Burston, 1991). He felt distant from his parents (Peebles, 2015). However, Funk (2006) explains that the father showed some tenderness towards his son and expressed his love through anxious care and ambivalent idealisation, while his mother idealised him fully, providing him with a strong self-consciousness that was anchored in the narcissistic love of the mother. This narcissistic motherly love might have resulted in an attitude that was later experienced by others as arrogance, especially in the 1930s and 1940s (Funk, 2006) and which might have influenced his idea that mental health is only possible through love, reason, creativity, independence, and a psychology of interpersonal relations (Cortina, 2024; Fromm, 1941, 1956; McLaughlin, 1998; Figure 2).

Fromm’s writing was, for many years, strongly influenced by his religious upbringing and the values which are attached to the Talmud (Akrap, 2011; Funk, 2000). His family expected him to carry on the strong tradition of becoming a religious leader (Hornstein, 2005). From 1918 he studied jurisprudence at the University of Frankfurt am Main and then moved in 1919 to Heidelberg to study sociology where he received a PhD in sociology in 1922 (Fromm, 1922). Although he became interested in Zionism (Widerström, 2013), he turned away from it soon after since it contradicted his idea of humanism (Lévy, 2002) which would later on become part of his explorations on love in The Art of Loving (1956).

His political ideas and interest came from Oswald Sussman, a young man who lived with the Fromm family in 1912–1914 since he helped the father with the wine business and engaged with Fromm in deep political discussions about Karl Marx and Friedrich Engels (Friedman, 2014) which became the foundation of his critical views on love, the society and capitalism (Durkin, 2025). Besides Fromm`s view that the society influenced love concepts (Durkin, 2025), he believed that love received from parents strongly influences an individual’s ability to love, in combination with the wish to become independent and autonomous, and all the psychic developments involved (Funk, 2006; Määtä & Uusiautti, 2013; Thompson, 2009).

### 4.2. Fromm’s first love and marriage (1924–1933)

From the mid-1920s, Fromm trained at the psychoanalytic sanatorium in Heidelberg and met Frieda Reichmann, who was 11 years his senior and who influenced his psychoanalytic ideas (Thompson, 2009). The two married in 1926 (Funk, 2006). When Fromm and Reichmann fell in love, he was her patient (Haffner-Marti & Hoffmann, 1998). He had an affair with her during the analysis and they finally married to keep their reputation (Chessick, 2002).

Throughout the love relationship, Frieda was described as controlling and as treating men, including Erich, as a child (Chessick, 2002). Although Erich had “impressed her by his genius”, however, the relationship did not last long (Hoffmann, 2011). They opened the “Frankfurt School of Psychoanalysis” in Germany in 1929 but separated in 1931 when Fromm became very ill with open tuberculosis (Funk, 2006). Frieda believed that his physiological illness was caused by psychological distress (Silver, 2000) while it could have also been a possibility for Fromm to remove himself from the relationship (Thompson, 2009). Thompson (2009) suggests that their love relationship was mainly influenced by Fromm’s unresolved issues with his mother. Only when he had freed himself from these issues could he end the relationship with Reichmann, which was connected to motherly love.

However, Fromm`s concept of love was not only influenced by his personal relationships in the 1920s. He was also strongly influenced by the societal changes in Germany (Frie, 2024, 2025). Fromm had a strong wish to understand human connection, based on the fact that he grew up during the First World War and experienced the rising of the Nazi regime in the 1920s (Frie, 2025; McLaughlin, 2023; New World Encyclopedia, 2025). He believed that love is an existential force that is needed to overcome the dichotomies of life and that it connects to existential needs to “escape isolation and anxiety” (Smith, 2020, p. 225). Love requires effort, maturity, the understanding of the individual within their contexts (Fromm, 1956). It is a force that counteracts war, cruelty and genocide and acts as a force of solidarity (Frie, 2024; Fromm, 1956). Frie (2024) points out that Fromm’s love concept was deeply influenced by his experiences in Nazi Germany and shows overlaps of defining the importance of human connections which his aunts Sofie Engländer and Gertrud Brandt who described these connections in their letters shortly before they were deported and killed in Germany. In particular, the idea of love as an important factor of mental health, well-being, and connectedness is relevant in Fromm’s work (1956), as well as in contemporary society (Yaden et al., 2017; Yaden et Newberg, 2022). Frie (2025) points out that Fromm’s study of freedom, the far-right, individual responsibility and agency are important aspects of his work on freedom and love that were informed during the rise of the Nazis.

### 4.3. Emigration and Fromm’s second marriage (1934–1950)

When Fromm and Reichmann moved to the USA in 1934, he got to know the psychoanalyst Karen Horney more intimately and the two built a relationship, which was influenced by rivalry and never led to marriage (Funk, 2006). It lasted until 1941 (Thompson, 2009).

Reichmann and Fromm only divorced in the USA in 1942. Being married to her had encouraged Fromm to explore further emotional dimensions of human relationships, as well as his interest in therapeutic human connections (Fromm-Reichmann, 1948; Thompson, 2009). The move to the United States impacted on Fromm strongly in terms of the post-war American socio-political atmosphere where Fromm talked about the “Pathology of Normalcy” (Fromm, 2010) and his critical views on how capitalism transformed individuals to abandon their existential human needs (Burston, 2024). Burston (2024) has argued that in these days, the United States need Fromm’s concept of humanism and love even more, based on the perception that present-day United States is even more alienated, fragile and atomized than in the mid 20th century.

Fromm has been described as a Neo-Freudian psychoanalyst, since he was very critical of several of Freud’s ideas, such as the Oedipus complex, the libido theory and Freud’s perspective on life and death instincts (Fromm, 1967). This is probably also a reason why Fromm was hardly recognised and cited in the United States in the 1960s and 1970s (Philipson, 2024) In an 1939 article, Fromm argues that individuals can love others and love themselves, while highlighting that selfishness is often confused with self-love (Fromm, 1939, 1958b). He suggests that without self-affirmation, one cannot genuinely affirm others (Fromm, 1939). Selfishness is merely a compensatory mechanism for an inner emptiness, and has little to do with love (Funk, 2019). In this way, Fromm collapses the false dichotomy between altruism and self-interest by reframing love of the self as a prerequisite for the love of others (Fromm, 1956), especially disagreeing with Freud’s ideas about self-love (Fromm, 1958b). In his theory of personality, which Fromm developed in the 1940s and beyond, he pointed out that humans are driven by two primary needs simultaneously: the need to be free and the need to belong (Fromm, 1941, 1947, 1955, 1956; Philipson, 2024). He emphasised the self-responsibility of each and every individual and analysed the social character as central to his clinical work (Layton, 2024).

Fromm’s *Escape from Freedom* (1941) positions love as a fundamental character trait which regulates the tension between autonomy and connection, a topic that was close to him with regard to his relationship with his mother. This represents the first shift towards his theory of freedom, belonging and love (Funk, 2000). Fromm was also deeply critical of capitalist society, arguing that while individuals were liberated from traditional social structures, systems and obligations, the rise of capitalism was responsible for feelings of powerlessness and isolation, a so-called “negative freedom” (Fromm, 1941). This gives rise to a love which is transactional, and rooted in consumption, dependence and status (Marcuse, 1964), a love concept that became increasingly popular after the Second World War. According to Fromm (1941), freedom to be oneself, or “positive freedom”, is achieved through love. Fromm’s (1941) idea of love in *Escape from Freedom* is better understood as an existential concept rather than a purely emotional one. He argues that love is

an active power in man; a power which breaks through the walls which separate man from his fellow men, which unites him with others; love makes him overcome the sense of isolation and separateness, yet it permits him to be himself, to retain his integrity (Fromm, 1941, p. 67).

Fromm distinguishes between authentic love and its substitutes. These substitutes include authoritarian love when one partner submits to the other, symbiotic fusion in which dependency inherits individuality, and narcissistic projection, where the love of the other is an extension of the love of the self (Fromm, 1941). It might be assumed that by writing this book, Fromm worked on the love relationship with his mother. Authentic love distinguishes itself through care, responsibility, respect and knowledge (Fromm, 1941), not through narcissistic love, as later developed by Fromm in *The Art of Loving* published in 1956 (Thompson, 2009; Funk, 2019; Funk, 2024).

Shortly after Fromm left Horney and divorced Reichmann, he got to know a German-born journalist, Henny Gurland, whom he married in 1944 (Thompson, 2009). They built a house together and Fromm seemed to have found the love of his life (Cherry, 2023). However, Gurland became bedridden without cure (Funk, 2006). Fromm cared for Gurland and moved with her in 1950 to Mexico to alleviate his wife’s illness and pain. In 1952, however, Fromm found Gurland dead in the bathroom where she had committed suicide (Funk, 2006).

### 4.4. Fromm’s third marriage and love concepts in selected books (1950–1970)

A few months after Gurland’s death, Fromm met Annis Freeman, an American woman whom he married in 1953 (Funk, 2006). According to Funk (2006), the loss of Gurland and the new love with Freeman prepared him to write *The Art of Loving* (1956). He felt that he had finally freed himself from his parental love and that love now meant that two people become one soul. He lived with Freeman in Mexico from 1955 to 1973; however, she contracted cancer soon after they met and Fromm retired in 1965 to help her fight the cancer (Funk, 2006).

Fromm’s time in Mexico was challenging in terms of differences in value sets and behaviour (Millán, 1995), but it also brought reflections about polarities of life and death, family and individuality (Fromm, 1964). He stayed in Mexico over 25 years and learned a lot about himself within the context of the differences of cultural setting (Fromm & Maccoby, 1970).

Fromm published his major works during his time in Mexico, where he became increasingly interested in a humane psychoanalysis and transformed from a strict and arrogant teacher to a humane and humorous one (Silva-García, 1992). Much of his work in Mexico was connected to being an analyst, a teacher, conference organiser and guest speaker (Millán, 1995; Silva-García, 1992). He focused increasingly on the conscious mind, the desire of self-actualisation, to reach one’s fuller potential and live authentically (Murphy, 2024).

During the 1950s, love becomes a central theme in his work, expressed in *The Art of Loving* (1956). There he argues that love is not a passive emotion that individuals experience but instead an active skill that needs to be learned and practised. According to Fromm, emotions such as love, envy, hatred, jealousy and greed come from different social structures (Fromm, 1955, 2024). This idea, explained in *The Sane Society* (1955) and in *For the Love of Life* (2024) was strongly connected to Fromm’s growing up and living during the First World War and the rise of the Nazi regime (Funk, 2000). If there is no love for humanity in the society and its structure, then love, sympathy and empathy cannot exist (Fromm, 2024).

Fromm’s love concept, as outlined in *The Sane Society* (1955), is both personal and social, situating love more firmly in the context of modern capitalist society. He centrally argues that the health of an individual is not separate from the health of the society they are in (Fromm, 1955). This assertion recontextualises love as both a structural necessity and an ethical imperative. Fromm (1955) begins by exploring social alienation as a symptom of modernity. Influenced by Marx’s notions of alienated labour, Fromm (1955) describes the estrangement of individuals in a capitalist society from their work, their community and themselves. Within this framework, love connects the individual to others in genuine, non-utilitarian ways, thereby countering the harms brought about by the capitalist system (Funk, 2019). Fromm’s experience of living in Germany, Switzerland, the USA and Mexico influenced his philosophical ideas in that he immersed himself in cross-cultural views of love, freedom and community (Friedman, 2014; Wehr, 2006).

Fromm (1955) is particularly concerned with the risk of love becoming commodified and treated as a form of exchange in capitalist society. Relationships, he argues, are often based on mutual gain, with a “marketing orientation” where individuals present themselves as commodities. This analysis reflects Fromm’s broader concern with what he terms the “pathology of normalcy” (Fromm, 1955). Modern societies often normalise attitudes which, from a humanistic point of view, are deeply pathological. In this sense, the commodification of love is made the norm, despite its effect on genuine emotional connection (Burston, 1991).

To resist this, Fromm proposes a “productive orientation”. Productive love requires the development of one’s own capacities as a human being and the recognition of the other as more than an object of consumption (Fromm, 1955). One of Fromm’s distinct contributions is that love is not limited to the bond between individuals but extends to a universal togetherness with humanity (Fromm, 1955). The absence of this solidarity, as in war or systemic inequality, is indicative of a society organised against love (Funk, 2000).

This social dimension of love which Fromm (1955) calls “brotherly love” values the dignity and humanity of others regardless of their utility, representing a recognition of the interconnectedness of all human beings. Having witnessed both World War I and the rise of the Nazi regime, Fromm (1994) later declares that societies lacking this love for humanity will produce structures devoid of sympathy and compassion. This insight is reflected in *The Sane Society,* where he warns that societies governed by greed and competition will inevitably end in violence and destruction (Fromm, 1955). Therefore, love becomes a collective necessity.

In *Escape from Freedom* (1941), Fromm had already laid the groundwork for understanding the dialectical relationship between freedom and love. This is expanded upon in *The Sane Society* by showing how love offers a constructive way to realise freedom without succumbing to isolation (Fromm, 1941). Love, in Fromm’s framework, is an expression of freedom, but not a freedom defined by detachment or independence. Rather, it is an expression of positive freedom: the ability to realise one’s self in connection with others (Fromm, 1955).

**Figure 3:**
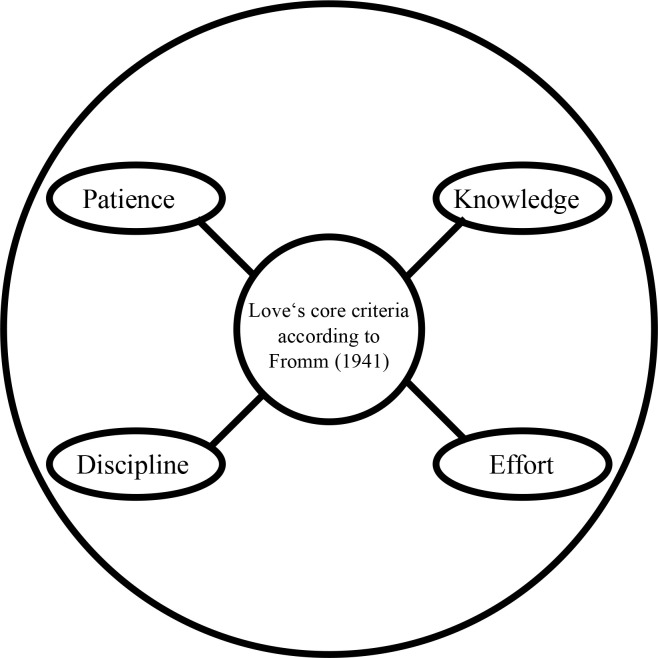
Love’s core criteria according to Fromm (1941) (authors’ own construction)

In *The Art of Loving*, Fromm (1956) begins by making the important distinction that if love is an art, then it requires knowledge and effort. Fromm (1956) questions the commonly held belief that love is something one “falls into” passively, stressing that love is a skill developed over time through knowledge, discipline, effort and patience (Figure 3). This redefinition is radical because, first, it frames love as a universal human capacity that extends beyond the personal feeling to all of humanity. Second, human agency is placed in the foreground since love is an active practice and not a passive experience (Burston, 1991).

**Figure 4:**
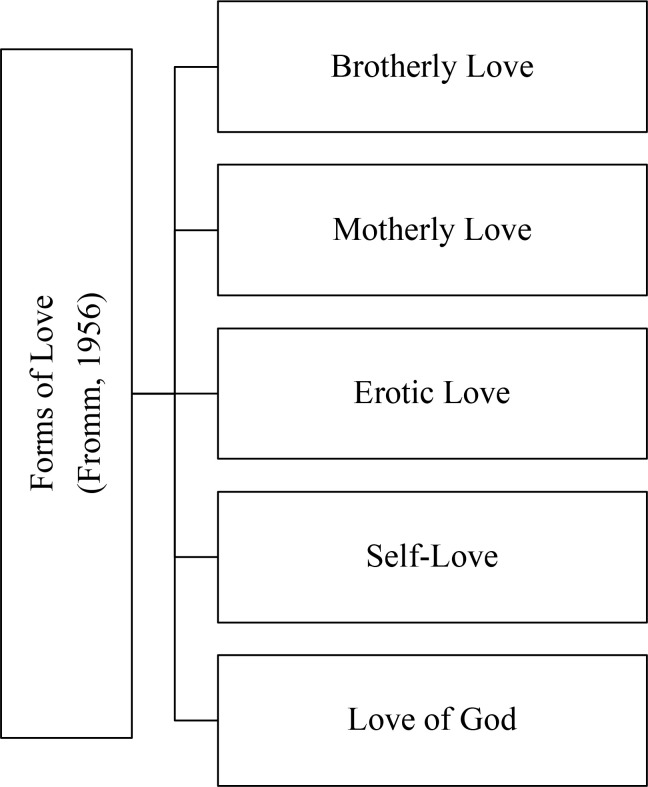
Forms of love according to Fromm (1956) (authors’ own construction).

Fromm (1956) identifies several distinct but interrelated forms of love, all of which derive from the same essential capacity for relatedness (Figure 4). Brotherly love is the foundational form of love, rooted in the solidarity among human beings. It is expressed in compassion, empathy, and the desire to alleviate suffering regardless of the personal utility of the other. Brotherly love encompasses humanity at large (Fromm, 1956).

Second, motherly love represents unconditional validation of the existence of another, affirming the existence of the child without requirement. However, Fromm cautions that motherly love must not hold the child back from becoming independent (Fromm, 1956). According to Funk (2006), Fromm needed many years to free himself from close motherly love and it took him many relationships to develop independence. One key experience must have been the death of his second wife in 1952. The archetypal mother loves the child based on the child’s existence and not to fulfil expectations. However, these role definitions in the context of love studies have been criticised, questioned and redefined. Conditional and unconditional love concepts cannot clearly be ascribed to mothers and fathers any longer owing to changing gender concepts (Haines & Schutte, 2022).

Third, erotic or romantic love is characterised by exclusivity and intensity (Fromm, 1956). Fromm warns against conflating erotic love with genuine, mature love (Excellence reporter, 2019). Erotic attraction alone is insufficient; it must be grounded in deeper commitment, respect and care (Fromm, 1956).

Fourth, contrary to the common perception that self-love is narcissistic, Fromm asserts that self-love is essential. Without affirming and respecting oneself, one cannot truly give love to others (Fromm, 1956). And finally, according to Fromm, the love of God expresses the human longing for unity with the ultimate or the transcendent, and is an expression of the human need to overcome isolation and alienation (Fromm, 1956).

One of Fromm’s most significant contributions is his conclusion that love requires maturity (Excellence Reporter, 2019). Based on his experiences with his parents and in his marriages, he identifies the four key elements of mature love as care, responsibility, respect and knowledge (Fromm, 1956). Care involves active concern for the life and growth of the other. Responsibility implies a readiness to respond to the needs of the beloved. Respect acknowledges the individuality and autonomy of the other, avoiding possessiveness. Finally, knowledge refers to the deep understanding of another person that goes beyond superficial traits. Only when all four elements are present can love be said to be genuine and sustaining.

For Fromm, a society that fails to cultivate love will be marked by authoritarianism, destructiveness and conformity (Fromm, 1956, 1973). Conversely, a society orientated to love will foster solidarity, creativity and freedom. In this sense, *The Art of Loving* is both a psychological treatise and a radical social critique (Funk, 2019). Fromm never stropped developing his theories and used his love relationships and marriages to develop further, especially during the 27 years with his third wife (Funk, 2006). In the 1960s he introduced the concept of biophilia as an expression of a love for life and all living beings (Fromm, 1964; Barbiero & Berto, 2021). Later, he characterised biophilia as a positive orientation towards life, contrasting it with the destructive tendency of necrophilia (Fromm, 1964). Fromm’s concept highlights love for nature as an ethical and moral orientation that encourages a responsible relationship with the environment. It is interpreted as a conscious striving for growth and vitality, closely linked to a moral obligation to protect and sustain nature (Fromm, 1964, 1976).

Fromm’s approach transcends emotional attraction to nature, positioning love for nature as an integral aspect of human values (Inomjon, 2020). This form of love reflects a productive and caring attitude that regards nature as a valuable counterpart and assumes responsibility for its preservation. Fromm presents biophilia as a foundation for altruistic behaviour and environmental engagement, which enhances personal well-being while strengthening the bond with nature and inspiring efforts to safeguard it (Gunderson, 2014).

### 4.5. Later life, unconditional love and loving (1970–1980)

In the last decade of his life, Fromm gained international recognition (Fromm, 2024). One of the ideas that remained with him until the end of his life was that people who love life are easily recognised by others, appear to be highly attractive, are loved by others and can be experienced as life lovers (Fromm, 2024). Accordingly, love, when it is unconditional and without purpose, is rare to find. However, this love is self-expressive, for which the act of loving is important itself, and is driven by loving and not by consuming love (Fromm, 2024). Real, authentic and mature love opens potentialities, develops productive tendencies and connects individuals across life and death through purpose and meaningfulness (He & Zhang, 2022).

## 5. Contemporary and Critical Voices on Fromm’s Concept of Love

Contemporary research has built upon Fromm’s ideas, increasingly emphasising the need for relatability, care, and mutual recognition in the face of social alienation. For instance, Zimovčáková (2013) revisits Fromm’s productive character, highlighting how one cannot truly love without knowledge of the other and without the active orientation of care. Similarly, Kristianti and Tantiani (2024) performed a qualitative study of Catholic widows in Indonesia, where participants described love in terms of giving, care and social responsibility. These descriptions align with Fromm’s productive conception of love, demonstrating the cross-cultural validity of Fromm’s theory (Zimovčáková, 2013).

Nevertheless, Fromm’s concept of love has been criticised for its applicability and precision (Smith, 2020). Smith (2020) critiques *The Art of Loving* from a theological and existential perspective, but his insights also apply to *Escape from Freedom,* questioning whether a broad, humanistic definition of love risks becoming overly abstract. Where Fromm cites discipline, faith and courage as requirements for love, critics argue that he provides little guidance on how individuals should go about cultivating these qualities in the context of a modern, complex society (Marcuse, 1964). Despite this, Fromm’s theory endures precisely because of its holistic vision of love as both psychological necessity and social remedy (Funk, 2019). Contemporary researchers on love have highlighted that love is a sociocultural concept, developing into an increasingly complex concept (Illouz, 2019) that is heteronormative and anthropocentric (Pettman, 2009). Saetra (2022) argues that human concepts build the basis for human–robot love, especially when it comes to building human–robot friendship, positive attention and care. However, human–robot love will lose out on altruistic and holistic love concepts with deep emotions. These trends indicate a moving away from concepts of love developed by Fromm and rather promote love that is built on self-interested and deficiency-orientated love (Saetra, 2022).

## 6. Conclusions and Recommendations for Future Theory and Practice

This article responded to the question of what love means in the context of Erich Fromm’s life and work. Fromm believed that humans suffer from an existential anxiety (angst) that can be counteracted through love. However, he believed that love is often misunderstood as a self-serving emotion that triggers possessiveness. He further assumed that real, mature love needs to bring flourishing, creativity and productivity, as well as encourage independence and virtue. Individuals only feel complete when they can love others fully, which then leads to the love of society and the world.

It seems plausible that Fromm’s love concepts were strongly influenced by his upbringing in a Jewish orthodox family, his religious beliefs and the experience of two world wars. He also developed his concepts of love through his experiences of three marriages and several other love relationships, their challenges and hardships. *The Art of Loving* (1956) was only written after the suicide of his second wife and the experience of new love with his third wife, which tremendously increased his vitality and love of life.

This article on Fromm’s life and love concepts can only provide possibly fruitful suggestions for future studies that might speak with greater precision and depth than does this work to the influence of specific events in Fromm's life on specific developments in his theorizing about love. Finally, Fromm’s ideas concerning love can be further explored with regard to new concepts of love and lived experiences in the context of contemporary society and contemporary conceptualisations of love regarding online love, long-distance love, technologised love and love beyond human and robotic love.

## Action editor

Lars-Gunnar Lundh served as action editor for this article.

## Open access

This article is distributed under the terms of the Creative Commons Attribution 4.0 International License (http://creativecommons.org/licenses/by/4.0/), which permits unrestricted use, distribution, and reproduction in any medium, provided you give appropriate credit to the original author(s) and the source, provide a link to the Creative Commons license, and indicate if changes were made.
